# Dynamics of *Plasmodium vivax* sporogony in wild *Anopheles stephensi* in a malaria-endemic region of Western India

**DOI:** 10.1186/s12936-017-1931-8

**Published:** 2017-07-11

**Authors:** Praveen Balabaskaran Nina, Ajeet Kumar Mohanty, Shuvankar Ballav, Smita Vernekar, Sushma Bhinge, Maria D’souza, Jayashree Walke, Suresh Kumar Manoharan, Anjali Mascarenhas, Edwin Gomes, Laura Chery, Neena Valecha, Ashwani Kumar, Pradipsinh K. Rathod

**Affiliations:** 1 Departments of Chemistry and of Global Health, University of Washington, Seattle, WA 98195 USA; 2 National Institute of Malaria Research, Field Unit, Campal, Goa 403001 India; 3 Goa Medical College and Hospital, Bambolim, Goa 403202 India; 4 National Institute of Malaria Research (ICMR), Sector 8, Dwarka, New Delhi 110077 India

**Keywords:** MESA-ICEMR, Goa, Vector infection, *Anopheles stephensi*, *Plasmodium vivax*, Oocysts, Sporozoites, Infection rate

## Abstract

**Background:**

In global efforts to track mosquito infectivity and parasite elimination, controlled mosquito-feeding experiments can help in understanding the dynamics of parasite development in vectors. *Anopheles stephensi* is often accepted as the major urban malaria vector that transmits *Plasmodium* in Goa and elsewhere in South Asia. However, much needs to be learned about the interactions of *Plasmodium vivax* with *An. stephensi.* As a component of the US NIH International Center of Excellence for Malaria Research (ICEMR) for Malaria Evolution in South Asia (MESA), a series of membrane-feeding experiments with wild *An. stephensi* and *P. vivax* were carried out to better understand this vector-parasite interaction.

**Methods:**

Wild *An. stephensi* larvae and pupae were collected from curing water in construction sites in the city of Ponda, Goa, India. The larvae and pupae were reared at the MESA ICEMR insectary within the National Institute of Malaria Research (NIMR) field unit in Goa until they emerged into adult mosquitoes. Blood for membrane-feeding experiments was obtained from malaria patients at the local Goa Medical College and Hospital who volunteered for the study. Parasites were counted by Miller reticule technique and correlation between gametocytaemia/parasitaemia and successful mosquito infection was studied.

**Results:**

A weak but significant correlation was found between patient blood gametocytaemia/parasitaemia and mosquito oocyst load. No correlation was observed between gametocytaemia/parasitaemia and oocyst infection rates, and between gametocyte sex ratio and oocyst load. When it came to development of the parasite in the mosquito, a strong positive correlation was observed between oocyst midgut levels and sporozoite infection rates, and between oocyst levels and salivary gland sporozoite loads. Kinetic studies showed that sporozoites appeared in the salivary gland as early as day 7, post-infection.

**Conclusions:**

This is the first study in India to carry out membrane-feeding experiments with wild *An. stephensi* and *P. vivax*. A wide range of mosquito infection loads and infection rates were observed, pointing to a strong interplay between parasite, vector and human factors. Most of the present observations are in agreement with feeding experiments conducted with *P. vivax* elsewhere in the world.

**Electronic supplementary material:**

The online version of this article (doi:10.1186/s12936-017-1931-8) contains supplementary material, which is available to authorized users.

## Background

*Plasmodium vivax* poses a serious threat to half of the world’s population, including the whole of South Asia [1]. In 2015, an estimated 13.8 million cases were caused by *P. vivax* [2]. India, Pakistan and Ethiopia are estimated to contribute greater than 80% of *P. vivax* cases in the world [2]. Hypnozoites, the dormant liver forms that are unique to *P. vivax* and *Plasmodium ovale,* can spontaneously re-activate and lead to periodic clinical relapses [3]. Many of the asymptomatic *P. vivax* infections in endemic areas are caused by hypnozoites [4] and these sub-clinical infections are difficult to detect, and thus complicate control measures.

Until now, in the absence of a culture system for continuous development of *P. vivax,* most of the knowledge on *P. vivax* is based on experimental and natural human infections [4–7]. For example, unlike *Plasmodium falciparum,* which invades all stages of erythrocytes, *P. vivax* selectively invades reticulocytes [6, 8–10]. *P. vivax* sexual development takes place in vectors when they ingest mature, infective gametocytes circulating in the peripheral blood [6, 11]. Compared to *P. falciparum*, gametocytes are more commonly observed in *P. vivax* infections [12], and they appear in blood smears much earlier in an infection [13]. Although clinical studies have shown that blood from asymptomatic, sub-microscopic gametocyte infections can infect mosquitoes, the correlation between *P. vivax* gametocytaemia and mosquito infections have been variable, with studies showing positive [14, 15], weak [5, 16, 17] and poor correlation [18–20]. Studying gametocytes in human blood is further complicated by the fact that the conventional microscopic techniques used for counting gametocytaemia are not always accurate and could contribute to the over- or underestimation of parasites in patient blood smears [21–24]. Furthermore, in controlled mosquito infections, the correlation between midgut oocyst numbers and salivary gland sporozoite load in *P. vivax* infections is not well understood. Successful mosquito infection of *Plasmodium* could be influenced by a number of parasite, vector and human factors including the state of gametocyte maturation, proportion of male and female gametocytes, variation in parasite genetics and transmission-blocking antibodies [11, 25–30]. Additionally, mosquito factors such as age, genetic diversity and microbiota could potentially affect the *P. vivax* infection rate and oocyst load [26, 31–33]. Many of the above uncertainties in studying the complex life cycle of *P. vivax* may be overcome with controlled feeding experiments.

The standard membrane feeding assay (SMFA) and direct skin feeding are the two most commonly used techniques for controlled mosquito infections studies [34]. Although direct skin feeding is more sensitive [34], ethical considerations and practical constraints favour the use of SMFA at many endemic sites.

As a part of the US NIH International Centers of Excellence for Malaria Research (ICEMR) [35–37], the Malaria Evolution in South Asia (MESA) programme has set up a controlled mosquito-feeding laboratory at the MESA-National Institute of Malaria Research (NIMR) joint study site in Panaji, Goa, India. For mosquito-feeding experiments, *P. vivax*-infected blood was obtained from patients recruited at the nearby Goa Medical College and Hospital (GMC), the other MESA study site in Goa [38]. To better understand the dynamics of parasite development in mosquitoes in southwestern India, 30 laboratory-feeding experiments were conducted with wild *An. stephensi* [39, 40] and *P. vivax*-infected patient blood. This paper describes the importance of patient blood parasitaemia and gametocytaemia on successful mosquito infection, as measured by oocyst numbers and sporozoite load. The potential role of Indian mosquito immunity in controlling *P. vivax* sporozoite load is also described.

## Methods

### Wild *An. stephensi* larvae collection and maintenance

Wild *An. stephensi* larvae and pupae were collected from breeding habitats, the curing waters in construction sites, around Ponda city in the state of Goa, India. The mosquito larvae and pupae were collected using the dipping technique and were transferred, along with breeding water, to plastic containers. The containers were then brought to the MESA insectary at the NIMR Goa field station. In the insectary, third and fourth instar larvae were separated from the first and second instar larvae and were reared separately. Pupae collected from the field were kept in 500-ml plastic bowls containing 200 ml of tap water, and inside a closed cage for controlled emergence. The larvae were reared in plastic trays containing tap water under laboratory conditions at 27 ± 2 °C, 70 ± 5% relative humidity and 12 h light/12 h dark photoperiod cycling. A pinch of Cerelac^®^ powder (Nestle) and fish food (1:1) mixture was given to the larvae once a day until pupal stage development was visible. Once formed, pupae were collected in fresh plastic bowls containing tap water, and then kept inside a closed cage for emergence of adults. The adult mosquitoes that emerged from these pupae were given 10% glucose soaked in a cotton pad. The species of emerged adult mosquitoes were identified using standard morphological keys.

### Ethics and approvals

The human subjects protocol governing collection of malaria parasites at GMC was approved by the Institutional Ethics Committee of Goa Medical College and Hospital, the University of Washington Institutional Review Board and by the US NIH/NIAID Division of Microbiology and Infectious Disease (DMID). The MESA-ICEMR programme project was additionally approved by the Health Ministry Screening Committee (HMSC) of the Government of India and by the Government of Goa Public Health Department.

### Blood collection from *P. vivax* patients

*Plasmodium vivax* patients confirmed by microscopy at GMC were briefed about the study by the project staff, and volunteers were recruited for the study. Prior to blood collection, informed consent was obtained from each patient. Approximately 6 ml of blood was drawn into an acid citrate dextrose vacutainer by venipuncture. Immediately after collection, the vacutainer was placed in a thermos flask maintained at 37 °C and was transported to the mosquito infection laboratory at NIMR-Goa.

### Mosquito-feeding experiments

Six to seven days old, adult, female mosquitoes were used for all the experiments. Female mosquitoes were caught using an aspirator and were transferred to plastic cups covered with mesh netting secured by a rubber band and a cup lid. Approximately 125 mosquitoes were placed in each cup. These mosquitoes were starved for 16–18 h prior to blood feeding. The close proximity of the malaria patient pool at GMC helped in receiving the patient blood at NIMR-Goa within one-and-a-half hours from the time of blood draw. Within minutes of arrival at NIMR-Goa, 2 ml of infected blood was added to a 5-cm wide water-jacketed membrane feeder fitted to a circulating water bath maintained at 37 °C. The feeder was positioned in the centre of the plastic container holding the mosquitoes. The blood was maintained at 37 °C during the entire 90-min feeding time to avoid premature exflagellation. After feeding, unfed mosquitoes were separated from fully engorged ones using an aspirator. The plastic cup containing the fully fed mosquitoes were kept in Percival incubators at 27 °C ±2 and 80% ±2 relative humidity. A cotton pad soaked in 10% glucose solution feed was provided until the mosquitoes were dissected at various time points (see below).

### Mosquito dissections and microscopy

For oocyst dissection on days 7/8 post blood feeding, five mosquitoes at a time were caught using a glass aspirator and were transferred to a test tube, which was then plugged with cotton. The test tube was placed in a −20 °C freezer for 2–3 min to immobilize the mosquitoes. Once the mosquitoes were knocked down, the test tube was placed on ice. Proboscis, wings and legs were first dissected to prevent accidental escape of infected mosquitoes. The dissected midgut was placed in a drop of 2% mercurochrome in PBS on a microscope slide with a coverslip, and examined for oocysts. The oocysts were counted using a phase contrast microscope at 5× and 10× (Carl Zeiss Axio Lab. A1). Salivary glands were dissected on different days post-infection, and were imaged at 40× magnification with a phase contrast microscope (Carl Zeiss Axio Lab. A1). Oocysts and sporozoites were counted independently by two project staff. The sporozoite load was calculated based on earlier studies [19, 41], and the gland index was recorded as: 1+ for (1–10 sporozoites), 2+ for (10–100 sporozoites), 3+ for (101–1000 sporozoites), 4+ for (>1000 sporozoites). In the present study, oocyst infection rate indicates the percentage of mosquitoes that contain one or more oocysts in a feeding experiment. Average oocyst load indicates the average number of oocysts in a population of mosquitoes in a feeding experiment. Sporozoite infection rate indicates the percentage of mosquitoes that contain one or more sporozoites in a feeding experiment.

### Patient blood parasite counts

Thin and thick smears were prepared with the donor patient blood and were stained with a 10% Giemsa solution. Patient blood parasitaemia and gametocytaemia in thin smears were counted by two trained technicians. Counting was done by the Miller reticule technique [42], and for every smear, 100 fields were counted. The ratio of large reticule to small reticule was calculated by ImageJ software, and was 4:1. The reticule factor was 25.

### Statistics

Statistical analysis was performed using the GraphPad Prism software. Pearson correlation was used to determine the significance of correlation between the counts of two independent slide readers. Linear regression was used to study the correlation between donor blood gametocytaemia and parasitaemia, and successful mosquito infections. The correlation between average oocyst load and sporozoite load was also evaluated using linear regression.

## Results

### Light microscopy

**Fig. 1:**
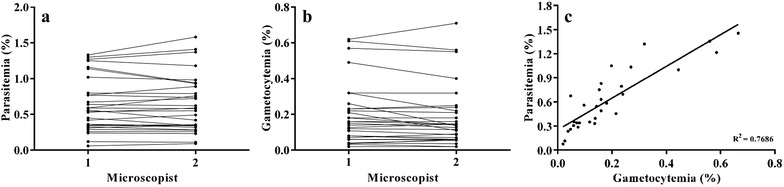
Assessment of co-relationship between gametocytaemia and parasitaemia in patient blood. **a**, **b** Graphs showing the direct comparison of individual values of parasitaemia and gametocytaemia counted by two microscopists to establish reproducibility and confidence in the data. **c** Linear regression to demonstrate the direct correlation between gametocytaemia and parasitaemia in patient blood

The parasitaemia and gametocytaemia counts (Fig. 1a, b) by two trained microscopists using the Miller reticule technique were significantly similar (parasitaemia-Pearson *r* 0.9685, p < 0.0001; gametocytaemia-Pearson *r* 0.9682, p < 0.0001). The average patient blood *P. vivax* parasitaemia (%) was 0.63 (Mean) ±0.38 (SD) and *P. vivax* gametocytaemia (%) was 0.19 (Mean) ±0.17 (SD), respectively. The number of female and male gametocytes were similar: The range and average of female gametocytes was 0.02–0.33% and 0.1 (Mean) ±0.08 (SD), respectively, and the range of male *P. vivax* gametocytes was 0–0.39%, and the average was 0.09 (Mean) ±0.09 (SD). The mean ratio of female to male *P. vivax* gametocytes was 1.31, and the range was 0–3.12. Parasite counts for each experiment from each of the two microscopists are given in Additional file 1. *P. vivax* gametocyte levels in patient blood were measured to study if it was the primary determinant of successful infection in mosquitoes. Linear regression analysis found a positive correlation between gametocytaemia and parasitaemia (R^2^ = 0.7686, p < 0.0001) (Fig. 1c). The average of the individual parasite counts of the two technicians were used for correlating gametocytaemia and parasitaemia to oocyst and sporozoite infection rates.

### Gametocytaemia, parasitaemia and correlation with infection

**Fig. 2:**
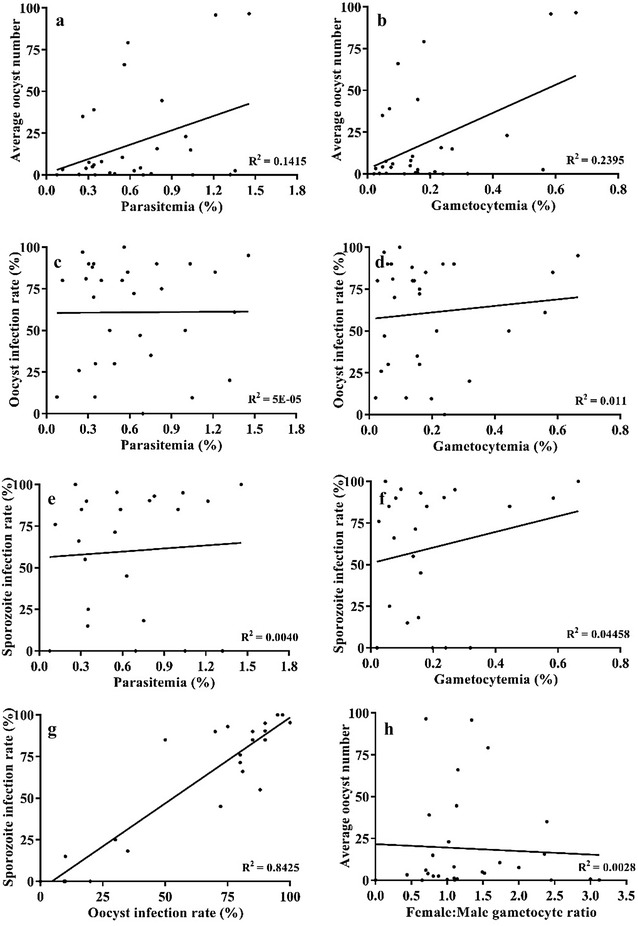
Assessment of the role of gametocytaemia, parasitaemia and gametocyte sex ratio on mosquito infection. **a**, **b** Correlation between parasitaemia/gametocytaemia and oocyst number. **c**, **d** Correlation between parasitaemia/gametocytaemia and oocyst infection rate. **e**, **f** Correlation between parasitaemia/gametocytaemia and sporozoite infection rate. **g** Correlation between oocyst and sporozoite infection rate. **h** Correlation between female: male gametocyte ratio and average oocyst number

Thirty feeding experiments were conducted with *P. vivax*-infected patient blood and wild *An. stephensi*. Detailed information on the number of mosquitoes dissected, infection rates, oocyst range, average oocyst load, and salivary gland index are given in Table 1. Weak correlations were observed between patient blood *P. vivax* parasitaemia and oocyst number (R^2^ = 0.14, p = 0.04) and between patient blood gametocytaemia and oocyst number (R^2^ = 0.24, p = 0.006) (Fig. 2a, b). The oocyst infection rate based on 30 feeds had no correlation with parasitaemia (R^2^ = 5E − 05, p = 0.97) and gametocytaemia (R^2^ = 0.01, p = 0.58) (Fig. 2c, d).

**Table 1:** Outcome of mosquito-feeding experiments

Exp. number	Patient blood smear (% of RBCs)			Mosquito Oocysts					Mosquito Sporozoites			
	*P. vivax*	Gametocytes		No. dissected	No. Pos.	Oocyst range	Av. oocyst no.	Infect. rate (%)	No. dissected	No. pos.	Infec. rate (%)	Gland index
		Male	Female									
1	1.35	0.31	0.25	23	14	0–13	2.5	61	5	2	40	3+ (2)
2	0.83	0.07	0.08	20	15	0–180	44.5	75	14	13	93	4+ (3), 3+ (10)
3	0.11	0.01	0.008	20	16	0–10	3.2	80	21	16	76	3+ (7), 2+ (8), 1+ (1)
4	0.07	0	0.02	20	2	0–1	0.1	10	20	0	0	NA
5	0.26	0.01	0.03	32	31	0–68	35	97	20	20	100	4+ (7), 3+ (12), 2+ (1)
6	0.28	0.04	0.03	16	13	0–17	4	81	21	14	66	3+ (8), 2+ (5), 1+ (1)
7	1	0.22	0.22	20	10	0–80	23	50	20	17	85	4+ (3), 3+ (13), 2+ (1)
8	0.69	0.14	0.09	20	0	0	0	0	19	0	0	NA
9	1.45	0.39	0.27	20	19	0–218	96.5	95	20	20	100	4+ (2), 3+ (18)
10	0.34	0.04	0.03	20	14	0–18	6	70	20	18	90	3+ (15), 2+ (2), 1+ (1)
11	0.35	0.03	0.08	20	2	0–2	0.15	10	20	3	15	3+ (1), 2+ (2)
12	1.03	0.15	0.12	20	18	0–62	14.9	90	20	19	95	3+ (15), 2+ (3), 1+ (1)
13	0.39	0.06	0.07	20	16	0–22	8	80	ND	NA	NA	NA
14	0.49	0.04	0.12	20	6	0–5	0.7	30	ND	NA	NA	NA
15	0.34	0.04	0.03	10	9	0–87	39	90	ND	NA	NA	NA
16	0.55	0.04	0.05	20	20	4–195	66	100	22	21	95	4+ (13), 3+ (8)
17	0.67	0.01	0.02	34	16	0–83	4.2	47	ND	NA	NA	NA
18	0.33	0.05	0.08	25	22	0–15	4.9	88	20	11	55	4+ (1), 3+ (7), 2+ (2), 1+ (1)
19	1.21	0.25	0.33	20	17	0–215	95.8	85	20	18	90	4+ (3), 3+ (14), 1+ (1)
20	0.79	0.07	0.16	20	18	0–40	15.7	90	21	19	90	4+ (3), 3+ (15), 1+ (1)
21	0.58	0.07	0.11	20	17	0–185	79.2	85	20	17	85	4+ (7), 3+ (9), 2+ (1)
22	0.3	0.01	0.03	20	18	0–22	7.6	90	20	17	85	4+ (2), 3+ (7), 2+ (7), 1+(1)
23	0.54	0.05	0.09	20	16	0–50	10.5	80	21	15	71	3+ (8), 2+ (7)
24	1.32	0.07	0.24	20	04	0–1	0.2	20	12	0	0	NA
25	1.05	0.09	0.1	21	02	0–2	0.1	10	20	0	0	NA
26	0.75	0.07	0.08	20	7	0–4	0.7	35	11	2	18	3+ (1), 2+ (1)
27	0.63	0.08	0.07	18	13	0–8	2.5	72	20	9	45	3+ (2), 2+ (5), 1+ (2)
28	0.35	0.03	0.03	20	6	0–2	0.3	30	20	5	25	3+ (1), 2+ (4)
29	0.45	0.1	0.11	14	7	0–4	1.2	50	ND	NA	NA	NA
30	0.23	0.01	0.01	27	7	0–3	0.3	26	ND	NA	NA	NA

Only experiments where at least 10 mosquitoes were dissected for salivary gland infections were used for correlating sporozoite infection rate with parasitaemia and gametocytaemia. Based on this criterion, experiments 1, 13, 14, 15, 17, 29 and 30 were not included for analysis

*ND* not done, *NA* not applicable

To calculate sporozoite infection rates, data from 23 of the 30 experiments, where at least ten mosquitoes were dissected for salivary gland infections, were included. Again, there was no correlation between sporozoite infection rate and patient blood parasitaemia (R^2^ = 0.004, p = 0.77) or patient blood gametocytaemia (R^2^ = 0.044, p = 0.33) (Fig. 2e, f). When oocyst and sporozoite infection rates were compared, a strong positive correlation (R^2^ = 0.84, p < 0.0001) was observed (Fig. 2g). The effect of sex ratio of female and male gametocytes on oocyst numbers was also compared, and no correlation was found (R^2^ = 0.0028, p = 0.78) (Fig. 2h). Interestingly, only five of the 30 patients had a female to male gametocyte sex ratio of >2, and in eight experiments where the average oocyst load was >20, the average sex ratio was 1.25. This indicates that higher oocyst load in wild *An. stephensi* in this region corresponds to a gametocyte sex ratio close to 1.

### Correlation between *P. vivax* oocyst and *P. vivax* sporozoite load in *An. stephensi*

**Fig. 3:**
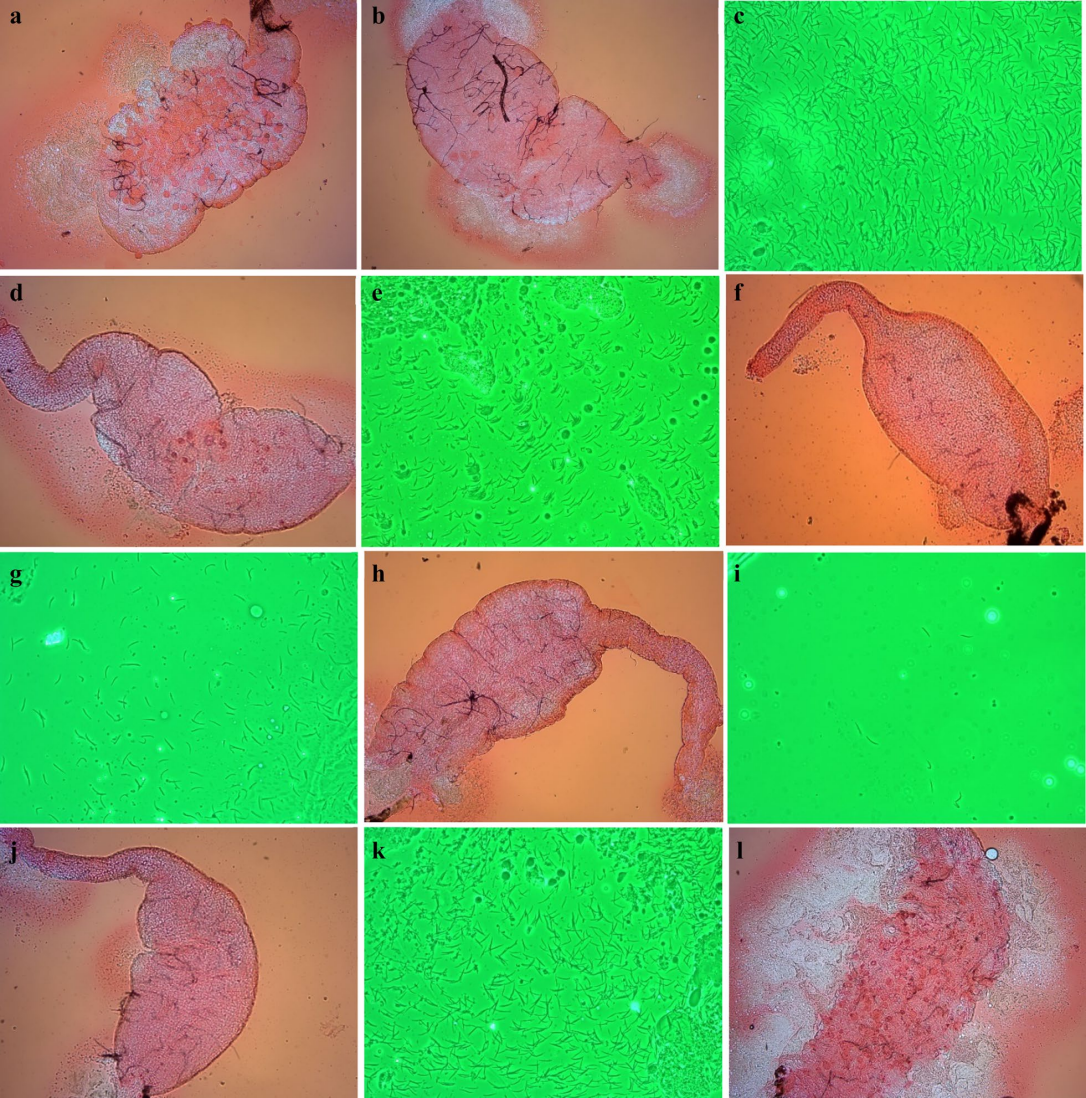
Oocyst and sporozoite infection in wild *An. stephensi.* Range of oocysts seen in a single feeding experiment (>200 oocysts: **a**; 0–10 oocysts: **b**). The midgut and gland indices of mosquitoes dissected on day 12/13 (**c** 4+, **e** 3+, **g** 2+, **i** 1+). The corresponding midguts of 4+, 3+, 2+, and 1+ mosquitoes are shown in **d**, **f**, **h**, and **j**. Midgut with heavily melanized and unhealthy oocysts, and corresponding gland with 3+ sporozoite load are shown in **l** and **k**, respectively. Melanized oocysts appear deformed, and contain dark pigments

**Fig. 4:**
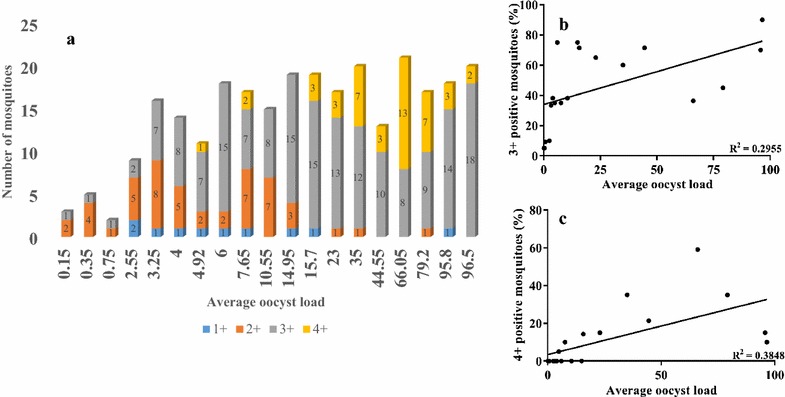
Correlation between oocyst and sporozoite load. **a** A stacked column comparing the average oocyst load (*horizontal axis*) to the sporozoite load (*vertical axis*) is shown. Sporozoite gland index are denoted by 1+ (*blue*), 2+ (*orange*), 3+ (*grey*), and 4+ (*yellow*). The number of positive mosquitoes are shown inside the *coloured bars*. **b**, **c** Linear regression to study correlation between average oocyst load and percentage of mosquitoes (out of total dissected) that had gland index of 3+ and 4+, respectively. Oocyst numbers were obtained on days 7/8 post feeding

In the *P. vivax* patient blood-feeding experiments, a wide range of oocyst numbers were observed in mosquitoes, even between individuals of the same batch. In some midguts, the number of oocysts were greater than 200 (Fig. 3a, b). The sporozoites were graded by gland index which ranged from 1+ to 4+. When salivary glands were dissected on days 12/13, the midguts were also examined for the presence of oocysts. The oocyst load on day 12/13 was significantly lower than on day 7 and the majority of the oocysts were not healthy. Images of varying loads of sporozoites (4+ to 1+) and their corresponding midguts are shown in Fig. 3c–j. A majority of the mosquitoes with a gland index of 4+ had some melanized and unhealthy oocysts in their midgut (Fig. 3c, d). In mosquitoes with gland index of 3+ , midguts with varying levels of oocysts from zero (Fig. 3e, f) to heavily loaded melanized and unhealthy oocysts were observed. There were no oocysts present in most of the mosquitoes that had a gland index of 2+ and 1+ (Fig. 3g–j). In several mosquitoes, midguts with a heavy load of unhealthy and melanized oocysts had a gland index of 3+ (Fig. 3l, k). Data from 19 positive salivary gland infection experiments, where at least ten mosquitoes were dissected (Table 1), allowed a comparison between average oocyst load and the sporozoite load. In general, there was a positive correlation as shown by the increase in mosquitoes with gland index of 3+ (R^2^ = 0.29, p = 0.016) and 4+ (R^2^ = 0.38, p = 0.004) (Fig. 4). Sporozoite loads of 4+ were seen even when the average oocyst load was less than 10 (Fig. 4). When the average oocyst load was greater than 79, the number of mosquitoes with gland index of 4+ decreased (Fig. 4), indicating a possible role of vector immune system in controlling *Plasmodium* infection.

### Kinetics *of P. vivax* sporozoites development at different time points post blood feeding in wild *An. stephensi*

The presence of *P. vivax* sporozoites in salivary glands was examined at different time points post-infection with patient blood. The first appearance of sporozoites was on day 7, and the sporozoite load in positive mosquitoes (two out of 17 dissected) were quite low (1+). The positivity of gland infection increased on day 8, and by day 12/13, the majority of oocyst had ruptured and salivary gland invasion was almost complete. Few mosquitoes were alive in the 4th week post blood feeding, and sporozoites were observed in 15 out of the 17 mosquitoes that were dissected. One mosquito survived up to day 30, and was also positive for salivary gland infection (Table 2).

**Table 2:** Sporozoites at different time points post blood feeding

Experiment no.^a^	Days post feeding										
	6	7	8	12/13	20	22	23	24	25	28	30
A				20/20	2/3	3/3		1/1			
B				21/22	3/4	5/5					
C				11/20	4/4					1/2	1/1
D			3/5	19/21					2/2		
E		1/6	2/6	17/20							
F	0/3	1/5	5/5	17/20			1/2				
G	0/5	0/6	3/5	15/21		2/2					

^a^Alphabetical numbering corresponds to experiment numbers 5, 16, 18, 20, 21, 22 and 23 given in Table 1. In these experiments, a few of the excess mosquitoes were sacrificed pre- and post- days 12/13 to examine the presence of sporozoites. Denominator indicates the number of mosquitoes dissected and numerator indicates number positive for sporozoites

## Discussion

Controlled laboratory-feeding experiments can help in understanding the development kinetics of *Plasmodium* in its vector [5, 6, 43]. Mosquito-feeding experiments were carried out using wild *An. stephensi* mosquitoes that emerged from field-caught larvae. In contrast, to date, most of the *P. vivax* infection experiments published earlier were done with colonized *Anopheles* mosquitoes [11, 14, 19, 41, 44–48]. When field mosquitoes are colonized, they generally undergo genetic drift, lose rare alleles, and show a decrease in heterozygosity and an increase in inbreeding that may not fully represent the biological interactions with parasite that happen in the wild [43, 49–51]. A recent report suggested that, by the 21st generation, colonized *Anopheles darlingi* underwent low to moderate differentiation from the original wild mosquito population [50]. Although collecting wild larvae requires additional human resources, is tedious, and collections are limited by seasonal availability, infection experiments with wild mosquitoes provide a solid baseline simulation of *Plasmodium*-*Anopheles* interactions in the wild. In the future, experiments with high passage colonized *An. stephensi* maintained in the MESA NIMR Goa insectary will be compared with the wild population for their susceptibility to *P. vivax* infections.

In many places, it is not common to have patient blood with *P. vivax* and vector laboratories in close proximity. The MESA-ICEMR mosquito infection laboratory at NIMR is located within 5 km of GMC, the study site where *P. vivax* patients are recruited and enrolled [38]. The close proximity of the two study sites facilitates easy transport of blood and enables feeding of mosquitoes to within one-and-a-half hours from the time of blood collection. The short duration between blood collection and mosquito feeding minimizes the loss in infectivity of the blood sample due to environmental factors, such as drop in temperature and change in blood pH [26, 52]. A recent study found that blood samples fed to mosquitoes at 8 h were significantly less infective than samples fed at 4 h post blood collection [26]. Furthermore, from the point of collection through mosquito feeding, the blood was always maintained at 37 °C to prevent premature exflagellation and any accompanying loss in mosquito infectivity.

Studying the correlation between the parasite load in the patient blood sample and the corresponding mosquito infections may help better understand the dynamics of parasite development in its vector in specific geographic locations. Correlations made between gametocyte density and mosquito infection based on thick smears are not always reliable, as up to 80% of gametocytes may be lost during the staining procedure [21], and the density of white blood cells (WBCs), to which gametocyte counts are related, is difficult to ascertain [22, 23]. In this study, two expert microscopists counted the parasites in 100 fields each by the Miller counting technique, and their counts were significantly similar, thereby giving greater confidence in the correlation analysis. Positive correlation was observed between gametocytaemia and parasitaemia in *P. vivax* patient smears. The correlation between gametocyte density and mosquito infection is often considered weak [7–10]. In the studies presented here, there was weak correlation between gametocytaemia/parasitaemia to oocyst numbers and no correlation to oocyst infection rate. Also, the ratio of female to male gametocytes did not affect mosquito infections. It appears that the number of mature/infective gametocytes in the blood sample is more important in determining the oocyst load than the absolute number of gametocytes. Furthermore, since the study was conducted in a malaria-endemic area, the transmission blocking immunity in the host serum [28–30] may also help determine the oocyst numbers. In experiments where the average mosquito oocyst load was greater than 20, the proportion of male gametocytes in patient blood was closer to 50%. This indicates that for good infectivity in this transmission setting, equal proportion of male and female gametocytes are required. In natural infections of *P. falciparum* [53–55] and in *P. vivax* [14], ratios of three or four female gametocytes to a male gametocyte is common, although there are variations depending on clones [56], treatment [57, 58] and the course of infection [59, 60]. The high proportion of male gametocytes in this geographical location may be an adaptation of the parasite to counter host’s transmission blocking immune mechanisms that may affect the production of male gametes [61]. Also, in *P. falciparum*, it has been suggested that gametocyte density may affect gametocyte sex ratios, with low density favouring greater proportion of male gametocytes and increased transmission [62].

Sub-microscopic gametocyte infections are less common with *P. vivax* than with *P. falciparum* [12] and, as reported earlier [6], gametocytes were seen in all of the patient samples. In agreement with earlier studies [19, 63], at one level, development of parasites from oocytes to sporozoites appeared very efficient in mosquitoes. There was a strong positive correlation between the percentage of mosquitoes positive for oocysts and per cent sporozoite positivity. However, the correlation between oocyst numbers in individual mosquitoes and sporozoite load was not linear. When the average oocyst load ranged between 0 and 66, a positive correlation with the sporozoite load was observed, as indicated by the increase in mosquitoes with gland indices of 3+ and 4+. While *P. vivax* studies of this type in *An. stephensi* are not known, an earlier study reported a linear correlation between oocyst and sporozoite load in *Anopheles dirus* and *Anopheles minimus* infected with *P. vivax* [47]. In the present study, however, when the average oocyst load was greater than 79, there was a decrease in the number of mosquitoes with a gland index of 4+. On day 12, some salivary glands were dissected in parallel with the midguts of the same mosquitoes and examined for oocyst health and load. In several cases, mosquitoes with numerous melanized and unhealthy oocysts failed to have a heavy sporozoite load (4+). This suggests that when the oocyst load is sufficiently high, enhanced melanization is activated in the mosquito, providing protection from tissue damage that would be caused by rupturing oocysts and continuously invading sporozoites. In contrast, an earlier study based on *P. vivax* infections in colonized *An. dirus* and *An. minimus* suggests that once oocysts are formed, the mosquito does not impose a ‘carrying capacity’ on the developing oocysts [47]. Here, in wild *An. stephensi*, the presence of melanized and unhealthy oocysts in the midgut strongly suggest a potential role of the mosquito’s immune system in controlling infection.

As seen in earlier studies [19, 41], in most feeding experiments, there was high variability in the oocyst load within individual mosquitoes of the same batch: Some were completely protected (zero oocysts) and some allowed large oocyst loads (>200). The genetic diversity of the wild mosquito population could be a critical factor that determines *P. vivax* infection load. *Anopheles gambiae*, the African malaria vector has been shown to exist in M and S forms based on its larval habitat and this adaptive divergence may influence the vector capacity and concomitant malaria epidemiology [64]. Future research on understanding the underlying traits that confer protection to *Plasmodium* infections in the wild would be of value.

## Conclusions

Goa is an endemic area for *P. vivax* and *An. stephensi* is the major malaria vector in this region. This study describes the findings of 30 feeding experiments carried out with wild *An. stephensi* and natural isolates of *P. vivax.* The present results point to important variations in wild *An. stephensi* to resist *P. vivax* infections. Additional, controlled infection studies will help further mimic, define, and understand the natural interactions of the parasite and the vector in the wild in South Asia.

## Additional file

**Additional file 1.** Parasite counts for each experiment from each of the two microscopists.

## Abbreviations

GMC: Goa Medical College and Hospital
MESA: Malaria Evolution in South Asia
ICEMR: International Center of Excellence for Malaria Research
NIMR: National Institute of Malaria Research
NIAID: National Institute of Allergy and Infectious Diseases
NIH: US National Institutes of Health
SMFA: standard membrane feeding assay
WBC: white blood cell
UW: University of Washington
DMID: Division of Microbiology and Infectious Disease
