# Supplementary material for: Dynamics of Plasmodium vivax sporogony in wild Anopheles stephensi in a malaria-endemic region of Western India
Source: Malar J. 2017 Jul 11;16:284. doi: 10.1186/s12936-017-1931-8 (PMC5504555; doi:10.1186/s12936-017-1931-8)
Supplement: Supplementary file 1 — Additional file 1. Parasite counts for each experiment from each of the two microscopists. [file 12936_2017_1931_MOESM1_ESM.docx]

**Supplementary Table 1. Parasite count by two technicians**

| **Sr. No.** | **Technician 1** | |  | **Technician 2** | |
| --- | --- | --- | --- | --- | --- |
|  | **Gametocytemia (%)** | **Parasitemia (%)** |  | **Gametocytemia (%)** | **Parasitemia (%)** |
| 1. | 0.55 | 1.41 |  | 0.57 | 1.3 |
| 2. | 0.14 | 0.89 |  | 0.18 | 0.77 |
| 3. | 0.02 | 0.11 |  | 0.032 | 0.12 |
| 4. | 0.02 | 0.09 |  | 0.02 | 0.06 |
| 5. | 0.055 | 0.26 |  | 0.04 | 0.26 |
| 6. | 0.07 | 0.28 |  | 0.08 | 0.29 |
| 7. | 0.4 | 0.98 |  | 0.49 | 1.02 |
| 8. | 0.25 | 0.72 |  | 0.23 | 0.67 |
| 9. | 0.71 | 1.58 |  | 0.62 | 1.33 |
| 10. | 0.09 | 0.34 |  | 0.07 | 0.34 |
| 11. | 0.115 | 0.34 |  | 0.12 | 0.36 |
| 12. | 0.22 | 0.93 |  | 0.32 | 1.14 |
| 13. | 0.126 | 0.34 |  | 0.15 | 0.45 |
| 14. | 0.11 | 0.42 |  | 0.21 | 0.56 |
| 15. | 0.06 | 0.32 |  | 0.08 | 0.36 |
| 16. | 0.086 | 0.59 |  | 0.108 | 0.529 |
| 17. | 0.06 | 0.76 |  | 0.036 | 0.59 |
| 18. | 0.146 | 0.35 |  | 0.126 | 0.31 |
| 19. | 0.56 | 1.18 |  | 0.61 | 1.25 |
| 20. | 0.24 | 0.79 |  | 0.23 | 0.798 |
| 21. | 0.18 | 0.58 |  | 0.18 | 0.59 |
| 22. | 0.057 | 0.283 |  | 0.06 | 0.326 |
| 23. | 0.147 | 0.55 |  | 0.14 | 0.54 |
| 24. | 0.32 | 1.37 |  | 0.32 | 1.27 |
| 25. | 0.136 | 0.94 |  | 0.26 | 1.16 |
| 26 | 0.146 | 0.734 |  | 0.16 | 0.77 |
| 27 | 0.16 | 0.62 |  | 0.16 | 0.64 |
| 28 | 0.06 | 0.355 |  | 0.06 | 0.35 |
| 29 | 0.21 | 0.49 |  | 0.22 | 0.42 |
| 30 | 0.036 | 0.23 |  | 0.04 | 0.24 |

**Supplementary Table 2. Male and female gametocyte counts**

| **Sr. No.** | **Technician 1** | |  | **Technician 2** | |
| --- | --- | --- | --- | --- | --- |
|  | **Male gametocyte (%)** | **Female gametocyte (%)** |  | **Male gametocyte (%)** | **Female gametocyte (%)** |
| 1. | 0.3 | 0.25 |  | 0.32 | 0.25 |
| 2. | 0.07 | 0.07 |  | 0.08 | 0.1 |
| 3. | 0.02 | 0 |  | 0.016 | 0.016 |
| 4. | 0 | 0.02 |  | 0 | 0.02 |
| 5. | 0.018 | 0.037 |  | 0.01 | 0.03 |
| 6. | 0.046 | 0.023 |  | 0.04 | 0.04 |
| 7. | 0.18 | 0.22 |  | 0.26 | 0.23 |
| 8. | 0.16 | 0.09 |  | 0.13 | 0.1 |
| 9. | 0.43 | 0.28 |  | 0.35 | 0.27 |
| 10. | 0.054 | 0.036 |  | 0.04 | 0.03 |
| 11. | 0.038 | 0.077 |  | 0.03 | 0.09 |
| 12. | 0.14 | 0.08 |  | 0.16 | 0.16 |
| 13. | 0.072 | 0.054 |  | 0.063 | 0.094 |
| 14. | 0.02 | 0.09 |  | 0.06 | 0.15 |
| 15. | 0.04 | 0.02 |  | 0.04 | 0.04 |
| 16. | 0.036 | 0.05 |  | 0.054 | 0.054 |
| 17. | 0.02 | 0.04 |  | 0.018 | 0.018 |
| 18. | 0.073 | 0.073 |  | 0.036 | 0.09 |
| 19. | 0.24 | 0.32 |  | 0.26 | 0.35 |
| 20. | 0.08 | 0.16 |  | 0.06 | 0.17 |
| 21. | 0.06 | 0.12 |  | 0.08 | 0.1 |
| 22. | 0.019 | 0.038 |  | 0.02 | 0.04 |
| 23. | 0.055 | 0.092 |  | 0.05 | 0.09 |
| 24. | 0.075 | 0.244 |  | 0.08 | 0.24 |
| 25. | 0.068 | 0.068 |  | 0.12 | 0.14 |
| 26. | 0.073 | 0.073 |  | 0.07 | 0.09 |
| 27. | 0.09 | 0.07 |  | 0.08 | 0.08 |
| 28. | 0.04 | 0.02 |  | 0.02 | 0.04 |
| 29. | 0.105 | 0.105 |  | 0.1 | 0.12 |
| 30. | 0.018 | 0.018 |  | 0.02 | 0.02 |
